# Aquaporins as Regulators of Cutaneous Malignancies: A Comprehensive Review

**DOI:** 10.3390/cells15050459

**Published:** 2026-03-04

**Authors:** Lara Camillo, Elisa Zavattaro, Paola Savoia

**Affiliations:** Department of Health Science, University of Eastern Piedmont, Via Solaroli 17, 28100 Novara, Italy; elisa.zavattaro@med.uniupo.it (E.Z.); paola.savoia@med.uniupo.it (P.S.)

**Keywords:** aquaporins, skin cancer, non-melanoma skin cancer, malignant melanoma, tumor microenvironment, membrane transport proteins

## Abstract

**Highlights:**

**What are the main findings?**

- Specific aquaporin isoforms show a layer-dependent expression in the skin and regulate hydration, cellular proliferation, inflammatory responses, and tissue homeostasis.
- Altered aquaporin expression and activity are linked to skin cancer development and progression, contributing to tumor growth, invasion, and aggressiveness in both non-melanoma skin cancers and malignant melanoma.

**What are the implications of the main findings?**

- Aquaporins act as key regulators of skin cancer biology, shaping tumor growth, invasion, and adaptation to microenvironmental stress.
- Defining isoform-specific aquaporin functions may facilitate the identification of novel therapeutic targets and guide future research in cutaneous oncology.

**Abstract:**

Aquaporins (AQPs) are a family of small integral membrane proteins that mediate the selective transport of water and, in some cases, small solutes such as glycerol and hydrogen peroxide. In the skin, distinct AQP isoforms are expressed throughout the epidermis, dermis, and hypodermis, where they play key roles in maintaining hydration, regulating keratinocyte and fibroblast proliferation, modulating inflammatory responses, and preserving overall tissue integrity. Increasing evidence indicates that aberrant AQP expression or function contributes to skin carcinogenesis, influencing tumor initiation, local invasion, metastasis, and responses to microenvironmental stress. Alterations in specific AQP isoforms have been associated with both major classes of cutaneous malignancies—non-melanoma skin cancers (NMSC), including basal cell carcinoma and squamous cell carcinoma, as well as malignant melanoma (MM)—yet their mechanistic contributions remain incompletely understood. This review synthesizes current knowledge on the involvement of each AQP isoform in skin cancer pathogenesis and progression, integrating findings from molecular, cellular, and in vivo studies. By clarifying the diverse roles of AQPs in cutaneous malignancies, this work aims to support the development of targeted interventions and guide future research in this rapidly evolving field.

## 1. Introduction

Aquaporins (AQPs) comprise a family of small integral membrane proteins that facilitate the passive, selective transport of water and small solutes such as glycerol, urea, and even hydrogen peroxide across cellular membranes [1]. In human beings, thirteen isoforms of AQPs (AQP0-AQP12) have been identified [2]. Based on their permeability to water and other molecules, AQPs are divided in three subtypes: orthodox (AQP0, 1, 4, 5, 6 and 8), aquaglyceroporins (AQP3, 7, 9 and 10) and unorthodox (AQP11 and 12) [3].

Beyond their role in fluid homeostasis, AQPs actively participate in the regulation of tissue integrity, cell metabolism, and response to stress [4,5,6,7]; on the other hand, defects in aquaporin function have been related to various disease conditions and pathological states [3,8].

In the skin, a variety of AQP isoforms are expressed across all major skin compartments, including the epidermis, dermis, hypodermis, adnexal structures (such as glands and hair follicles), and vascular elements. For instance, studies report that isoforms such as AQP3 are predominantly localized in keratinocytes—especially those in the basal and spinous layers—where AQP3-mediated glycerol transport is essential for epidermal hydration, barrier function, stratum corneum formation, and skin elasticity [9]. Other isoforms (e.g., AQP9) are enriched in the upper epidermal layers and are thought to contribute to keratinocyte differentiation as well as water and solute transport [9].

Beyond physiological homeostasis, emerging evidence points to a role for AQPs in cutaneous tumorigenesis. The aberrant expression or regulation of AQPs has been observed in both major categories of skin cancer: non-melanoma skin cancers (NMSC)—including basal cell carcinoma (BCC) and squamous cell carcinoma (SCC)—and malignant melanoma (MM) [10].

For instance, immunohistochemical analyses on a series of human skin tumor biopsies revealed the consistent strong expression of AQP3 across BCC, SCC, and melanoma samples, whereas AQP1 was generally absent in tumor cells but detected in surrounding stroma and neovessels. On the other hand, in a cohort of 58 melanoma patients, the expression patterns of AQP1, AQP8, and AQP9 were associated with prognostic parameters (e.g., mitotic index, sentinel lymph node status, Breslow thickness, ulceration), highlighting a potential link between specific AQP expression profiles and clinical outcome [10].

Experimental evidence indicates that AQPs contribute to tumor cell survival, migration, and resistance to therapy. In the melanoma cell line WM266.4, the overexpression of AQP3 and AQP9 increased resistance to arsenite-induced apoptosis, likely via the downregulation of p53 and upregulation of anti-apoptotic proteins (e.g., Bcl-2, XIAP) [11].

These observations suggest that altered AQP expression might facilitate tumor progression not only by modulating fluid and solute handling, but also by contributing to metabolic adaptation, oxidative stress response, and anti-apoptotic signaling.

Given these observations, a comprehensive synthesis of the existing data on AQP expression and function in skin—under normal and pathological (cancer) conditions—is timely and potentially valuable. A better understanding of how specific AQP isoforms influence tumor initiation, progression, invasion, metastasis, and therapy resistance could reveal new biomarkers and therapeutic targets and guide future experimental and clinical research efforts.

## 2. Materials and Methods

The design of the present study was based on the Preferred Reporting Items for Systematic Reviews and Meta-Analyses (PRISMA) protocol [12]. A comprehensive literature search was performed in the main electronic databases, including PubMed, Scopus, Web of Science, and Cochrane, to identify studies relevant to this review. The search strategy combined keywords such as “Aquaporins”, “Skin Cancer”, “Melanoma”, “Non-Melanoma Skin Cancer”, “Cutaneous Tumors”, and “Skin Neoplasms”, using Boolean operators to refine the search. Boolean operators were applied as follows: “AND” to include articles containing all terms, “OR” to include articles containing any of the synonyms, and “NOT” to exclude irrelevant topics.

**Figure 1:**
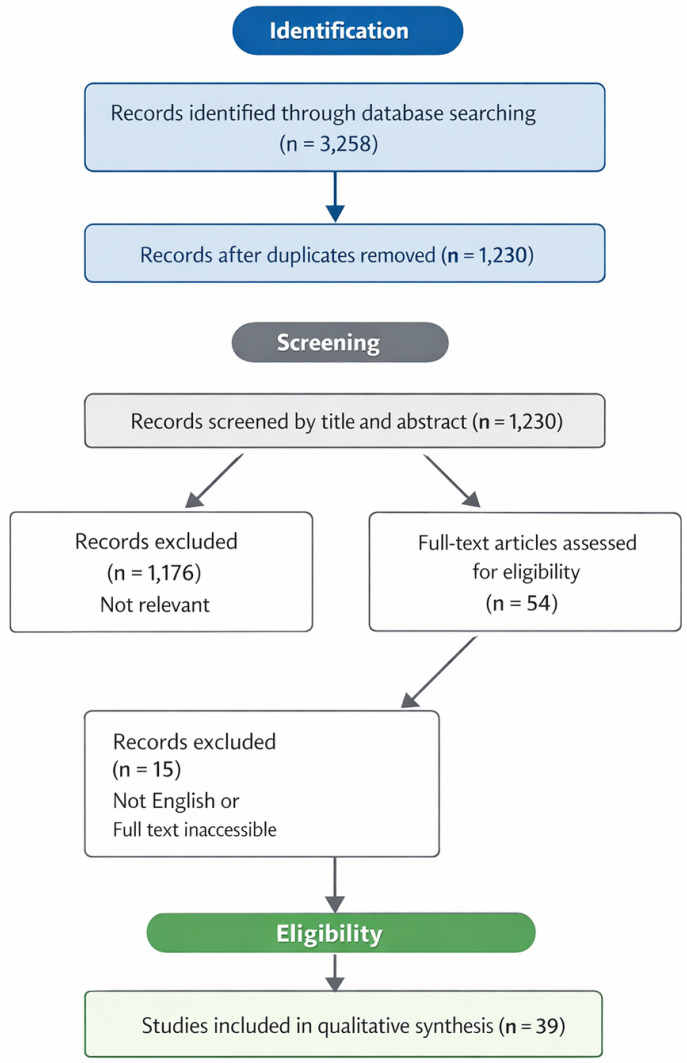
Flow chart of study selection. From 3258 articles, 1230 were screened; 1176 excluded for relevance and 15 for language or full-text issues. In total, 39 studies were included in the review.

The initial search retrieved 3258 articles. After removing duplicates, 1230 unique records remained for screening. Titles and abstracts were carefully evaluated, leading to the exclusion of 1176 articles that were not directly relevant to the topic. An additional 15 articles were excluded because they were either not written in English or were inaccessible in full text (Figure 1).

Ultimately, 39 studies met all inclusion criteria and were thoroughly analyzed to support the synthesis presented in this review.

## 3. Aquaporins in Normal Skin

### 3.1. Expression Patterns

In the skin, eight AQP isoforms have been identified (AQP1, 3, 5, 7, 8, 9, 10, 11), which are differently distributed throughout the epidermis, dermis and hypodermis [13].

AQP1 has been detected in the dermis, expressed by the endothelial cells of dermal capillaries and fibroblasts, and in the epidermis in melanocytes of the basal layer [14,15].

AQP3 is mainly localized in keratinocytes of the basal and spinosus layers, specifically in the plasma membrane and intracellular compartment [16], as well as in sebaceous glands [10,17,18].

AQP5 is found in eccrine sweat gland cells, in particular on the apical and basolateral plasma membranes of clear cells, whereas in ductal components and apocrine glands it is absent [19]. AQP5 has also been revealed in the stratum granulosum of the palmar epidermis, showing a strong expression in the plasma membrane of keratinocytes [20,21].

AQP7 is expressed by adipocytes of the hypodermis, Langerhans cells and dermal dendritic cells [9,17].

AQP9 has been detected in the stratum granulosum of the epidermis [22], as well as in cultured differentiated keratinocytes [17,22].

Lastly, AQP10 is expressed in the stratum corneum of the epidermis [13,23], but little is known about its role in the epidermis beyond the transport of water and glycerol throughout the epidermis [14].

### 3.2. Physiological Roles

The main function of AQPs in the skin is to maintain proper hydration; however, it has been demonstrated that they are involved also in other fundamental cell processes [14]. AQP1, which is expressed in vascular endothelial cells, exchanges water between blood vessels and the dermis, guaranteeing skin hydration [13,14]. In addition, AQP1 has been demonstrated to contribute to the cell migration of both endothelial cells and keratinocytes [13]. Finally, AQP1 seems to be upregulated in the presence of hypertonic stress in both melanocytes and fibroblasts [14,24,25].

AQP3 is fundamental for the transport of glycerol and water from the base of the epidermis to the stratum corneum [26], eventually contributing to the maintenance of skin hydration [17,27]. In addition, AQP3 is involved in the wound healing process, favoring keratinocyte migration and proliferation, and finally inducing keratinocyte differentiation [17,28,29].

AQP5 appears to play a supporting role in the palmar epidermis by maintaining the proper hydration fundamental for resistance to strong mechanical stress [21].

AQP7 contributes to the hypertrophy of hypodermal adipocytes [17], to the primary immune response, and to cell migration in the epidermis [9].

AQP9, similarly to AQP3, contributes not only to proper skin hydration, but also to the wound healing process and keratinocyte differentiation [9,22].

## 4. Aquaporins in Skin Cancer

### 4.1. Non-Melanoma Skin Cancer

Non-melanoma skin cancers (NMSCs)—which include BCC and SCC—are the most common malignancies worldwide, with a rising incidence driven by increased ultraviolet exposure and aging populations. Although these tumors generally grow slowly and have a low metastatic potential, they can cause significant local tissue damage and morbidity [30]. At the molecular level, the dysregulation of pathways controlling proliferation, apoptosis, and stress responses underlies NMSC development and progression [31]. Emerging evidence also points to a functional contribution of AQPs to NMSC pathobiology, highlighting their potential as diagnostic markers and therapeutic targets.

#### 4.1.1. AQP1

AQP1 expression has been investigated in numerous solid tumors, including adenoid cystic carcinoma, bladder, breast, cervical, colon, colorectal, hepatocellular, lung, ovarian, prostate, renal cell carcinoma and mesothelioma [32], and is generally associated with an increased risk of tumor progression and poor prognosis. For example, Lehnerd et al. [33] reported that, in a cohort of 107 consecutive oropharyngeal squamous cell carcinoma cases, AQP1 expression was observed exclusively in a subgroup of aggressive basaloid-like tumors. Similarly, an immunohistochemical analysis performed on 50 primary esophageal squamous cell carcinomas [34] showed that AQP1 was primarily located in the cytoplasm and/or the nuclear membrane of carcinoma cells, with patients exhibiting “cytoplasm-dominant” AQP1 expression displaying a significantly lower 5-year survival rate (47.1%) compared to other patients (83.2%). Despite these findings in other tumor types, information on AQP1 expression and its clinical relevance in NMSCs remains very limited. According to the literature, the only study addressing this issue is that by Osorio et al., who performed an immunohistochemical analysis on 15 BCC and 15 SCC specimens. AQP1 was not detected in tumoral cells in either BCC or SCC but was observed in the surrounding stromal and vascular compartments, and, in SCC, also in infiltrating lymphocytes. These findings are consistent with those observed in other tumor types and support a potential role for AQP1 in tumor growth. Furthermore, its expression in peritumoral lymphocytes and fibrotic areas suggests an involvement in tumor-associated inflammation and immune surveillance.

#### 4.1.2. AQP3

AQP3, in particular, has been extensively studied in skin cancer, but the findings are sometimes conflicting. Strong expression has been reported in the basal keratinocytes of human SCC samples (*n* = 40) [35] and in an induced mouse model of SCC. Notably, AQP3-deficient (AQP3^−/−^) mice exhibited a resistance to cancer development, showing reduced epidermal cell proliferation, which is a fundamental step for cancer development, together with a reduced ATP content and glycerol and glucose levels [35], highlighting the role of AQP3 in the development of SCC. Similarly, Wang et al. [36] have found that, in actinic keratosis (AK, *n* = 12), Bowen’s disease (BD, *n* = 15) and SCC (*n* = 20) samples, AQP3 expression was significantly higher compared with normal skin and was localized in the plasma membrane. Moreover, AQP3 was moderately expressed in SCC nests and horn pearls. These results confirm the hypothesis that AQP3 is fundamental for skin carcinogenesis progression [36]. Another study conducted by Niu et al. [18] reported that, in solar keratosis (*n* = 24), BD (*n* = 26) and SCC (*n* = 43), AQP3 was expressed at both the gene and protein level, whereas in BCC (*n* = 32) and MM (*n* = 28) AQP3 was absent. These results are in agreement with their previous work [37]. Voss et al. [38] have demonstrated that AQP3 was absent in almost all BCC specimens (*n* = 13), while in SCC samples (*n* = 5) its expression was “patchy”, presenting a lower protein expression at the borders of the lesion. Moreover, they found that AQP3 downregulation was correlated with Ki67 expression, hypothesizing that AQP3 has anti-proliferative properties and suggesting that its downregulation could be a marker of tumorigenic potential [38]. Other studies, however, report contrasting results. Ramadan et al. [39] observed a significantly reduced expression of AQP3 in AK (*n* = 30) and SCC (*n* = 30) biopsies compared with normal skin, showing a “patchy” immunohistochemical stain in SCC cells. They have also found that, in BCC (*n* = 33), the expression of AQP3 is similar to healthy controls [39]. In partial agreement with these results, Seleit et al. [40] have found that 66.7% of BCC (*n* = 30) and 93.3% of SCC (*n* = 30) specimens analyzed were positive for AQP3 immunohistochemical stain, despite the intensity of protein expression being lower compared with normal skin. Additionally, they reported the translocation of AQP3 from the cell membrane to the cytoplasm, which may favor cell survival [40]. Collectively, these studies indicate a complex and context-dependent role for AQP3 in skin carcinogenesis, with expression patterns varying by tumor type and stage.

### 4.2. Melanoma

Cutaneous melanoma is a malignant tumor originating from melanocytes and represents one of the most aggressive forms of skin cancer. Although less common than non-melanoma skin cancers, its incidence has been steadily rising worldwide, largely due to increased ultraviolet (UV) exposure and changes in lifestyle [41]. Melanoma is characterized by a high metastatic potential and significant mortality, making the early detection and understanding of molecular mechanisms critical. The dysregulation of pathways controlling cell proliferation, apoptosis, and immune evasion contributes to melanoma initiation and progression, and emerging evidence suggests that aquaporins (AQPs) may play a functional role in these processes, potentially influencing tumor growth, invasion, and resistance to therapy [10,42].

#### 4.2.1. AQP1

The study published by Osorio et al. reported a strong expression of AQP1 in all examined common melanocytic nevi (*n* = 5), whereas normal melanocytes and atypical cells from dysplastic naevi (*n* = 10) showed no detectable expression. Also, all tested melanoma cases were negative for AQP1 in tumor cells, although the protein was present in small neovessels, peritumoral stroma, and melanophages surrounding the tumor. The reason for this apparent discrepancy in AQP1 expression between normal melanocytes and melanoma cells observed in this study remains unknown. Nevertheless, its presence in vascular endothelial cells within the tumor microenvironment suggests a potential role in promoting tumor growth and metastatic spread through neovascularization.

Further clinical evidence supports a role for AQP1 in melanoma progression. Imrédi et al. [43] examined AQP1 expression in 78 melanoma patients and found that 66.7% were positive. Significantly higher AQP1 H-scores were observed in patients classified as “high-risk”, and AQP1 expression was significantly associated with BRAF v600 mutations, whereas no correlations were found with other established prognostic markers, such as Breslow thickness, Clark level and mitotic index. In contrast, Camillo et al. [44] observed AQP1 expression in 25% of Superficial Spreading Melanoma (*n* = 44), while all Nodular Melanoma cases were negative, suggesting a possible histotype-specific pattern. The potential impact of AQP1 on melanoma progression and patient outcomes was further supported by a subsequent study by the same author [45], involving 67 metastatic melanoma patients. Patients with brain metastases—and consequently reduced survival—exhibited a significantly higher AQP1 expression in the primary tumor, when compared with patients with extracranial secondary localizations. Interestingly, AQP1 expression in brain metastases was lower and more heterogeneous than in primary lesions, with a higher expression in melanoma cells located farther from capillaries, suggesting a possible hypoxia-driven regulation of this protein. These observations are consistent with the findings in mouse melanoma models, where the silencing of AQP1 led to reduced neovessel formation and impaired tumor progression [46,47].

#### 4.2.2. AQP3

Several studies have suggested a potential role for AQP3 in melanoma biology. In vitro, the overexpression of AQP3 in melanoma cell lines has been shown to enhance resistance to oxidative stress and chemotherapeutic agents, likely through the modulation of glycerol metabolism and activation of anti-apoptotic signaling pathways [42]. In addition, Gao et al. [11] demonstrated that AQP3 can inhibit the therapeutic effect of arsenite by upregulating the expression of antiapoptotic genes, such as *Bcl-2* and *XIAP*, while downregulating proapoptotic genes, including *P53* and *Bax*, further supporting a potential role in chemoresistance. Despite these mechanistic insights, the role of AQP3 in melanoma progression remains controversial. For instance, Lugassy et al. [48] reported that AQP3 is downregulated in primary melanomas with an angiotropic profile, suggesting that reduced AQP3 expression may facilitate a switch toward extravascular, neural crest-like migratory behavior rather than directly marking metastatic potential. Similarly, Niu et al. observed a negative AQP3 expression in all 28 melanoma samples analyzed, whereas focal AQP3 positivity was detected in 8 of 26 benign nevi. In contrast, another study [10] demonstrated a strong AQP3 expression in both benign and atypical melanocytic nevi, as well as in all exanimated melanomas. These apparently conflicting results highlight the complexity of AQP3 regulation in melanocytic lesions and suggest that its role may be context-dependent, varying with tumor stage, microenvironment, or methodological differences across studies. Notably, none of these studies established a clear correlation between AQP3 expression and patients’ clinical outcomes, which could account for some of the observed discrepancies.

#### 4.2.3. AQP5

The role of AQP5 in melanoma remains poorly defined. In vitro studies on human melanoma cell lines have reported AQP5 expression in a subset, suggesting a potential involvement in tumor cell biology [11]. However, analyses of clinical melanoma samples indicate that AQP5 is largely absent in patient tumors, contrasting with its detectable expression in certain benign melanocytic nevi [44]. This discrepancy between in vitro and ex vivo observations may reflect differences in the tumor microenvironment, methodological sensitivity, or inherent biological heterogeneity. Overall, these findings suggest that, while AQP5 may be expressed under experimental conditions, its relevance in human melanoma pathophysiology requires further investigation using larger patient cohorts.

#### 4.2.4. AQP8 and AQP9

AQP8 and AQP9 expression and their possible clinical correlation were evaluated in our previous study [44]. In a cohort of 58 melanoma patients, including 14 Nodular Melanomas and 44 Superficial Spreading Melanomas, AQP8 was positive in 50% (7/14) of Nodular and 77% (34/44) of Superficial Spreading samples, while AQP9 was positive in 57.1% (8/14) and 83.7% (37/44), respectively. AQP8 expression was significantly associated with a negative sentinel lymph node biopsy, suggesting a favorable prognostic implication, whereas AQP9 expression correlated with a lower Breslow thickness and the absence of ulceration. Furthermore, the analysis of disease-free survival over an 8-year follow-up period demonstrated that patients with a positive AQP expression had a better prognosis. These findings suggest that AQP8 and AQP9 may serve as potential prognostic markers in melanoma, with their expression linked to less aggressive tumor features and improved disease-free survival. However, these preliminary data still need to be validated and confirmed in larger cohorts, particularly because they appear to conflict with other findings.

### 4.3. Functional Implications in Tumor Biology

The involvement of AQPs in skin cancer progression has been demonstrated by several studies, as mentioned above. However, the molecular mechanisms through which AQPs contribute to skin carcinogenesis are still unclear, since the available evidence in the literature is limited and mainly focused on melanoma. For instance, AQP1 seems to promote the migration of the mouse melanoma cell line B16F10, leading to lung metastasis when injected into animals [49]. On the other hand, Saadoun et al. [50] have demonstrated that, in AQP1-null mice, the development of subcutaneous melanoma was slowed due to impaired angiogenesis, eventually leading to enhanced survival, thus demonstrating the fundamental role of this AQP in tumor growth. Camillo et al. [51] showed that, 24 h after UVB irradiation, melanoma cell line A375 presented higher levels of AQP1, 8 and 9 expression in dimeric form, which was associated with increased ROS levels, an altered cell cycle and a reduced cell viability.

AQP3 knockdown in two melanoma cell lines—MNT-1 and A375—led to decreased H_2_O_2_ and glycerol influx together with lower ROS levels, demonstrating the pivotal role of AQP3 in maintaining the cell redox balance [42]. Furthermore, AQP3 silencing resulted in reduced cell adhesion, proliferation and migration, mainly due to the reduced glycerol transport and lipid synthesis, which impacted ATP production [42]. The overexpression of AQP3 and AQP9 in human melanoma cell line WM266.4 inhibits arsenite-induced apoptosis by downregulating p53 and Bax and activating Bcl-2, thereby contributing to melanoma chemoresistance [11].

In normal keratinocytes, the interaction of AQP3 with Notch1 controls cell differentiation [52]. Nevertheless, in keratinocyte cancer cell lines, the expression of both AQP3 and Notch1 receptors is disrupted, indicating an indirect role of AQP3 in the suppression of cancer growth [35,52].

### 4.4. AQPs as Pharmacological Targets

In recent years, AQPs have garnered significant attention not only for their physiological functions, but also as potential therapeutic targets, especially for neoplastic diseases [53]. Various molecules have been proposed as AQP inhibitors, including acetazolamide, anti-epileptic drugs, mercury and heavy metal ion inhibitors, small molecules and monoclonal antibodies [54], although most of them are limited to in vitro or in vivo studies [53,55,56]. The search for novel AQP-targeting drugs is challenging due to the high structural similarity among AQP isoforms, their widespread distribution in tissues, and both conceptual and technical obstacles [53,55]. Nevertheless, important efforts have been made by researchers, even in the field of skin cancer. Several studies have demonstrated that gold- and copper-based compounds can efficiently inhibit AQP3, leading to the reduced cell proliferation and migration of both melanoma and keratinocyte tumoral cell lines. Indeed, da Silva et al. [42] have demonstrated that the inhibition of AQP3 mediated by three gold compounds and Auphen impaired the cell proliferation, migration and adhesion of two melanoma cell lines—MNT1 and A375—linked to a reduction in glycerol flux. Another study conducted by Pimpão et al. [57] has proven that the inhibitory effect mediated by polyoxotungstates (POTs) on AQP3 drastically reduces MNT1 growth and migration, suggesting their potential role as an anti-cancer drug. Similarly, Nave et al. [58] have shown that Cuphen, a copper-based compound, efficiently inhibited AQP3 activity, resulting in the reduced cell viability of MNT1, human epidermoid carcinoma A431, human keratinocyte (HaCaT) and murine melanoma B16F10 cell lines in both free form or incorporated in liposomes.

Another promising target is AQP1. Indeed, Nicchia et al. [46] and Simone et al. [47] have demonstrated that the inhibition of AQP1 through siRNA silencing led to reduced tumor growth and metastasis formation in a mouse model of melanoma, mainly due to impaired angiogenesis. Although data on AQP agonists and antagonists remain limited, these findings suggest that AQPs could serve as viable targets for isoform-specific therapeutic interventions, paving the way for future drug discovery efforts.

## 5. Conclusions

Collectively, the available evidence highlights aquaporins as key regulators of skin physiology and important contributors to skin cancer biology. Beyond their fundamental role in maintaining epidermal hydration and tissue homeostasis, specific AQP isoforms—particularly AQP1, AQP3, AQP8, and AQP9—appear to influence tumor-related processes such as cell proliferation, migration, angiogenesis, oxidative stress regulation, and resistance to apoptosis. Although expression patterns and functional roles vary across tumor types and study models, altered AQP regulation emerges as a recurring feature in both non-melanoma skin cancers and melanoma. The observed associations between AQP expression profiles and clinical or prognostic parameters further support their potential utility as biomarkers and therapeutic targets. However, inconsistencies across studies underscore the need for standardized methodologies and larger, well-characterized patient cohorts. Future research should focus on clarifying isoform-specific mechanisms, tumor-stage dependencies, and microenvironmental influences to better define the clinical relevance of AQPs in skin cancer prevention, diagnosis, and treatment.

## Data Availability

The original contributions presented in this study are included in the article. Further inquiries can be directed to corresponding author.

## Abbreviations

The following abbreviations are used in this manuscript:

NMSC	Non-Melanoma Skin Cancer
BCC	Basal Cell Carcinoma
SCC	Squamous Cell Carcinoma
AQP	Aquaporin

## References

[1] Brown D. (2017). The Discovery of Water Channels (Aquaporins). Ann. Nutr. Metab..

[2] Bhend M.E., Kempuraj D., Sinha N.R., Gupta S., Mohan R.R. (2023). Role of aquaporins in corneal healing post chemical injury. Exp. Eye Res..

[3] Azad A.K., Raihan T., Ahmed J., Hakim A., Emon T.H., Chowdhury P.A. (2021). Human Aquaporins: Functional Diversity and Potential Roles in Infectious and Non-infectious Diseases. Front. Genet..

[4] Login F.H., Nejsum L.N. (2023). Aquaporin water channels: Roles beyond renal water handling. Nat. Rev. Nephrol..

[5] Ahmed T., Ghafoor S. (2021). Aquaporins; Systemic, Functional and Therapeutic Correlations in Health and Disease. J. Pak. Med. Assoc..

[6] Dutta A., Das M. (2022). Deciphering the role of aquaporins in metabolic diseases: A mini review. Am. J. Med. Sci..

[7] Vrettou C.S., Issaris V., Kokkoris S., Poupouzas G., Keskinidou C., Lotsios N.S., Kotanidou A., Orfanos S.E., Dimopoulou I., Vassiliou A.G. (2024). Exploring Aquaporins in Human Studies: Mechanisms and Therapeutic Potential in Critical Illness. Life.

[8] Bi Y., Pang S., Liu Y., Cheng J., Ma Q., Song A., Yin X., Wang J. (2025). Aquaporins in lipid metabolism: Functions and regulation in health and disease. Lipids Health Dis..

[9] Gunia-Krzyżak A. (2025). Aquaporins in the Skin: Molecular Regulators of Hydration and Potential Targets for Cosmetic Applications. Cosmetics.

[10] Osorio G., Zulueta-Dorado T., González-Rodríguez P., Bernabéu-Wittel J., Conejo-Mir J., Ramírez-Lorca R., Echevarría M. (2019). Expression Pattern of Aquaporin 1 and Aquaporin 3 in Melanocytic and Nonmelanocytic Skin Tumors. Am. J. Clin. Pathol..

[11] Gao L., Gao Y., Li X., Howell P., Kumar R., Su X., Vlassov A.V., Piazza G.A., Riker A.I., Sun D. (2011). Aquaporins mediate the chemoresistance of human melanoma cells to arsenite. Mol. Oncol..

[12] Page M.J., McKenzie J.E., Bossuyt P.M., Boutron I., Hoffmann T.C., Mulrow C.D., Shamseer L., Tetzlaff J.M., Akl E.A., Brennan S.E. (2021). The PRISMA 2020 statement: An updated guideline for reporting systematic reviews. BMJ.

[13] Karimi N., Ahmadi V. (2024). Aquaporin Channels in Skin Physiology and Aging Pathophysiology: Investigating Their Role in Skin Function and the Hallmarks of Aging. Biology.

[14] Patel R., Kevin Heard L., Chen X., Bollag W.B., Yang B. (2017). Aquaporins in the Skin. Aquaporins.

[15] Mobasheri A., Marples D. (2004). Expression of the AQP-1 water channel in normal human tissues: A semiquantitative study using tissue microarray technology. Am. J. Physiol. Physiol..

[16] Bollag W.B., Aitkens L., White J., Hyndman K.A. (2020). Aquaporin-3 in the epidermis: More than skin deep. Am. J. Physiol. Physiol..

[17] Hara-Chikuma M., Verkman A. (2008). Roles of Aquaporin-3 in the Epidermis. J. Investig. Dermatol..

[18] Niu D., Bai Y., Yao Q., Hou W., Zhou L., Huang X., Zhao C. (2021). Expression and Significance of AQP3 in Cutaneous Lesions. Anal. Cell. Pathol..

[19] Iizuka T., Suzuki T., Nakano K., Sueki H. (2011). Immunolocalization of aquaporin-5 in normal human skin and hypohidrotic skin diseases. J. Dermatol..

[20] Blaydon D.C., Lind L.K., Plagnol V., Linton K.J., Smith F.J., Wilson N.J., McLean W.I., Munro C.S., South A.P., Leigh I.M. (2013). Mutations in AQP5, Encoding a Water-Channel Protein, Cause Autosomal-Dominant Diffuse Nonepidermolytic Palmoplantar Keratoderma. Am. J. Hum. Genet..

[21] Blaydon D.C., Kelsell D.P. (2014). Defective channels lead to an impaired skin barrier. J. Cell Sci..

[22] Sugiyama Y., Yamazaki K., Kusaka-Kikushima A., Nakahigashi K., Hagiwara H., Miyachi Y. (2014). Analysis of aquaporin 9 expression in human epidermis and cultured keratinocytes. FEBS Open Bio.

[23] Tricarico P.M., Mentino D., De Marco A., Del Vecchio C., Garra S., Cazzato G., Foti C., Crovella S., Calamita G. (2022). Aquaporins Are One of the Critical Factors in the Disruption of the Skin Barrier in Inflammatory Skin Diseases. Int. J. Mol. Sci..

[24] Leitch V., Agre P., King L.S. (2001). Altered ubiquitination and stability of aquaporin-1 in hypertonic stress. Proc. Natl. Acad. Sci. USA.

[25] Boury-Jamot M., Sougrat R., Tailhardat M., Le Varlet B., Bonté F., Dumas M., Verbavatz J.-M. (2006). Expression and function of aquaporins in human skin: Is aquaporin-3 just a glycerol transporter?. Biochim. Biophys. Acta (BBA) Biomembr..

[26] Verdier-Sévrain S., Bonté F. (2007). Skin hydration: A review on its molecular mechanisms. J. Cosmet. Dermatol..

[27] Hara-Chikuma M., Verkman A. (2005). Aquaporin-3 functions as a glycerol transporter in mammalian skin. Biol. Cell.

[28] Hara-Chikuma M., Verkman A.S. (2007). Aquaporin-3 facilitates epidermal cell migration and proliferation during wound healing. J. Mol. Med..

[29] Hara-Chikuma M., Takahashi K., Chikuma S., Verkman A.S., Miyachi Y. (2009). The expression of differentiation markers in aquaporin-3 deficient epidermis. Arch. Dermatol. Res..

[30] Samarasinghe V., Madan V. (2012). Nonmelanoma skin cancer. J. Cutan. Aesthetic Surg..

[31] Didona D., Paolino G., Bottoni U., Cantisani C. (2018). Non Melanoma Skin Cancer Pathogenesis Overview. Biomedicines.

[32] Moosavi M.-S., Elham Y. (2019). Aquaporins 1, 3 and 5 in Different Tumors, their Expression, Prognosis Value and Role as New Therapeutic Targets. Pathol. Oncol. Res..

[33] Lehnerdt G.F., Bachmann H.S., Adamzik M., Panic A., Köksal E., Weller P., Lang S., Schmid K.W., Siffert W., Bankfalvi A. (2015). AQP1, AQP5, Bcl-2 and p16 in pharyngeal squamous cell carcinoma. J. Laryngol. Otol..

[34] Yamazato Y., Shiozaki A., Ichikawa D., Kosuga T., Shoda K., Arita T., Konishi H., Komatsu S., Kubota T., Fujiwara H. (2018). Aquaporin 1 suppresses apoptosis and affects prognosis in esophageal squamous cell carcinoma. Oncotarget.

[35] Hara-Chikuma M., Verkman A.S. (2008). Prevention of Skin Tumorigenesis and Impairment of Epidermal Cell Proliferation by Targeted Aquaporin-3 Gene Disruption. Mol. Cell. Biol..

[36] Wang X., Tao C., Yuan C., Ren J., Yang M., Ying H. (2017). AQP3 small interfering RNA and PLD2 small interfering RNA inhibit the proliferation and promote the apoptosis of squamous cell carcinoma. Mol. Med. Rep..

[37] Niu D., Kondo T., Nakazawa T., Yamane T., Mochizuki K., Kawasaki T., Matsuzaki T., Takata K., Katoh R. (2012). Expression of aquaporin3 in human neoplastic tissues. Histopathology.

[38] Voss K.E., Bollag R.J., Fussell N., By C., Sheehan D.J., Bollag W.B. (2011). Abnormal aquaporin-3 protein expression in hyperproliferative skin disorders. Arch. Dermatol. Res..

[39] Ramadan W.M., Gheida S.F., El-Ashmawy A.A., Shareef M.M. (2017). Aquaporin-3 Expression in Common Hyperproliferative Skin Disorders: An Immunohistochemical Study. J. Egypt. Women’s Dermatol. Soc..

[40] Seleit I., Bakry O.A., Al Sharaky D., Ragheb E. (2015). Evaluation of Aquaporin-3 Role in Nonmelanoma Skin Cancer: An Immunohistochemical Study. Ultrastruct. Pathol..

[41] Gieniusz E., Skrzydlewska E., Łuczaj W. (2024). Current Insights into the Role of UV Radiation-Induced Oxidative Stress in Melanoma Pathogenesis. Int. J. Mol. Sci..

[42] da Silva I.V., Pimpão C., Paccetti-Alves I., Thomas S.R., Barateiro A., Casini A., Soveral G. (2024). Blockage of aquaporin-3 peroxiporin activity by organogold compounds affects melanoma cell adhesion, proliferation and migration. J. Physiol..

[43] Imrédi E., Tóth B., Doma V., Barbai T., Rásó E., Kenessey I., Tímár J. (2016). Aquaporin 1 protein expression is associated with BRAF V600 mutation and adverse prognosis in cutaneous melanoma. Melanoma Res..

[44] Camillo L., Esposto E., Gironi L.C., Airoldi C., Alhamed S.A., Boldorini R.L., Zavattaro E., Savoia P. (2023). Aquaporin 1, Aquaporin 8, and Aquaporin 9 Expressions in Malignant Melanoma: A Possible Correlation with Prognosis and Clinical Outcome. J. Clin. Med..

[45] Imrédi E., Liszkay G., Kenessey I., Plotár V., Gödény M., Tóth B., Fedorcsák I., Tímár J. (2018). Aquaporin-1 Protein Expression of the Primary Tumor May Predict Cerebral Progression of Cutaneous Melanoma. Pathol. Oncol. Res..

[46] Nicchia G.P., Stigliano C., Sparaneo A., Rossi A., Frigeri A., Svelto M. (2012). Inhibition of aquaporin-1 dependent angiogenesis impairs tumour growth in a mouse model of melanoma. J. Mol. Med..

[47] Simone L., Gargano C.D., Pisani F., Cibelli A., Mola M.G., Frigeri A., Svelto M., Nicchia G.P. (2017). Aquaporin-1 inhibition reduces metastatic formation in a mouse model of melanoma. J. Cell. Mol. Med..

[48] Lugassy C., Lazar V., Dessen P., Oord J.J.v.D., Winnepenninckx V., Spatz A., Bagot M., Bensussan A., Janin A., Eggermont A.M. (2011). Gene expression profiling of human angiotropic primary melanoma: Selection of 15 differentially expressed genes potentially involved in extravascular migratory metastasis. Eur. J. Cancer.

[49] Hu J., Verkman A.S. (2006). Increased migration and metastatic potential of tumor cells expressing aquaporin water channels. FASEB J..

[50] Saadoun S., Papadopoulos M.C., Hara-Chikuma M., Verkman A.S. (2005). Impairment of angiogenesis and cell migration by targeted aquaporin-1 gene disruption. Nature.

[51] Camillo L., Esposto E., Gironi L.C., Zavattaro E., Savoia P. (2025). Nicotinamide Counteracts Ultraviolet-B-Induced Cytotoxic Effects and Aquaporins Overexpression in the A375 Melanoma Cell Line. Dermato.

[52] Guo L., Chen H., Li Y., Zhou Q., Sui Y. (2013). An Aquaporin 3-Notch1 Axis in Keratinocyte Differentiation and Inflammation. PLoS ONE.

[53] Verkman A.S., Anderson M.O., Papadopoulos M.C. (2014). Aquaporins: Important but elusive drug targets. Nat. Rev. Drug Discov..

[54] Pimpão C., Wragg D., da Silva I.V., Casini A., Soveral G. (2022). Aquaglyceroporin Modulators as Emergent Pharmacological Molecules for Human Diseases. Front. Mol. Biosci..

[55] Abir-Awan M., Kitchen P., Salman M.M., Conner M.T., Conner A.C., Bill R.M. (2019). Inhibitors of Mammalian Aquaporin Water Channels. Int. J. Mol. Sci..

[56] Tradtrantip L., Jin B.-J., Yao X., Anderson M.O., Verkman A.S., Yang B. (2017). Aquaporin-Targeted Therapeutics: State-of-the-Field. Aquaporins.

[57] Pimpão C., da Silva I.V., Mósca A.F., Pinho J.O., Gaspar M.M., Gumerova N.I., Rompel A., Aureliano M., Soveral G. (2020). The Aquaporin-3-Inhibiting Potential of Polyoxotungstates. Int. J. Mol. Sci..

[58] Nave M., Castro R.E., Rodrigues C.M., Casini A., Soveral G., Gaspar M.M. (2016). Nanoformulations of a potent copper-based aquaporin inhibitor with cytotoxic effect against cancer cells. Nanomedicine.

